# The immune microenvironment in tumors: focus on canine and feline spontaneous neoplasms

**DOI:** 10.3389/fvets.2025.1674694

**Published:** 2025-10-28

**Authors:** Maddalena Rizzi, Giulia D’Annunzio, Chiara Tugnoli, Giuseppe Sarli, Luisa Vera Muscatello

**Affiliations:** ^1^Department of Veterinary Medical Sciences, University of Bologna, Bologna, Italy; ^2^Experimental Zooprophylactic Institute of Lombardy and Emilia-Romagna “Bruno Ubertini”, Brescia, Italy

**Keywords:** tumor microenvironment, spontaneous neoplasms, TILs, TAMs, immune checkpoints

## Abstract

Companion animals develop spontaneous tumors with biological and immunological features closely resembling human cancers. The tumor microenvironment (TME), particularly its immune infiltrates, plays a pivotal role in tumor progression and immune evasion. This review summarizes current knowledge on the composition and function of immune cells (including T cells, B cells, macrophages, dendritic cells, neutrophils, and mast cells) in the TME of canine and feline tumors. A better understanding of these mechanisms may aid in identifying prognostic biomarkers and novel immunotherapeutic targets in both veterinary and human oncology.

## 1 Introduction

The tumor microenvironment (TME) is a complex and dynamic network of cells that infiltrates and surrounds the tumor. Its interaction with neoplastic cells plays a pivotal role in shaping tumor behavior, influencing progression, malignancy, and therapeutic response. While this relationship is well documented in human oncology, it remains an emerging area of investigation in veterinary medicine.

Understanding the contribution of different immune cell populations within spontaneous tumors in dogs and cats offers promising perspectives for the development of novel therapeutic strategies aimed at treating or slowing tumor progression.

The TME refers to the non-malignant cellular context that surrounds the neoplasm and actively interacts with it, shaping key aspects of tumor biology such as progression, metastasis, and response to therapy (1). It is composed of blood vessels, fibroblasts (also known as tumor-associated stromal cells), immune cells including lymphocytes and myeloid-derived inflammatory cells, signaling molecules, and is further characterized by the presence of an extracellular matrix (ECM) that provides structural and biochemical support (2, 3). The specific composition of TME plays a crucial role in shaping the defining traits of cancer, known as the hallmarks of cancer. These include resistance to cell death, persistent proliferative signaling, evasion of growth suppressors, activation of invasive and metastatic processes, uncontrolled cell proliferation, and the induction of angiogenesis (2–5).

In recent years, the immune component of the TME has gained considerable attention for its ability to support and restrain tumor growth. Key players in modulating cancer development and progression include immune cells such as tumor-associated macrophages (TAMs), dendritic cells (DCs), regulatory T cells (Tregs), cytotoxic CD8 + T cells and myeloid-derived suppressor cells (MDSCs). These cell populations display remarkable functional plasticity in their pro- or anti-tumorigenic roles.

This review provides a critical overview of recent literature on the topic, aiming to clarify the intricate interactions within the TME and explore the dual role of the innate and adaptive immune systems in promoting and suppressing tumor development.

In veterinary oncology, investigating the TME is becoming increasingly important as a step towards improving cancer diagnosis, prognosis, and treatment in companion animals. Cancers in dogs and cats often present with high biological and clinical heterogeneity, and their immune microenvironments can greatly influence disease progression and therapeutic response. Nevertheless, the immunological landscape of spontaneous tumors in veterinary species is less well characterized than that of human neoplasms. The characterization of immune infiltrates—such as TAMs, T lymphocytes, and myeloid-derived suppressor cells—as well as immune evasion mechanisms involving checkpoint molecules such as PD-1, PD-L1, and CTLA-4, is beginning to transform our approach to cancer therapy in animals. Leveraging knowledge from human immuno-oncology and adapting it to the veterinary context enables clinicians and researchers to develop more personalized and effective therapeutic strategies, ultimately improving outcomes for animal and human patients alike. The comparative lens is reinforced by ECM immune convergence: collagen signatures and TAM–ECM phenotypes track with outcome across dogs, cats, and humans, emerging as shared hallmarks that can enable cross-species biomarkers and inform combination therapies integrating immune checkpoint blockade with stroma-targeted approaches (6–8).

## 2 Extracellular matrix—immune cross talk

The ECM plays a key role in shaping the immune environment within tumors by affecting how innate immune cells activate, differentiate, and survive, especially in human cancers such as pancreatic ductal adenocarcinoma (PDAC) and breast cancer (9). Its physical properties, including stiffness and density, directly influence immune cell behavior and contribute to the immunosuppressive tumor microenvironment (9).

For example, macrophages respond to ECM stiffness; when cultured in dense, collagen-rich matrices, they exhibit enhanced T cell suppression and reduced recruitment of CD8 + T cells, as shown in experimental models (10). Furthermore, elevated collagen levels in human breast and colorectal cancers are associated with poor prognosis and increased metastatic potential. This is partly because collagen binds to LAIR-1, an inhibitory receptor expressed on immune cells such as natural killer (NK) and T cells (11). Engagement of LAIR-1 by collagen inhibits cytotoxic immune responses, thereby facilitating tumor immune evasion (11). Additionally, tumor cells themselves may produce transmembrane and extracellular collagens, amplifying this immunosuppressive signal within the TME (9).

Beyond its mechanical properties, the ECM undergoes proteolytic remodeling by enzymes like those from the ADAM and ADAMTS families. This process releases matrikines—bioactive ECM fragments with immunomodulatory functions. For instance, in human colorectal cancer, cleavage of the ECM proteoglycan versican (VCAN) produces versikine, which promotes the differentiation of conventional dendritic cells that enhance T cell-mediated anti-tumor immunity (12).

In veterinary oncology, VCAN proteolysis by ADAMTS enzymes generates versikine, a bioactive fragment enriched at the invasive fronts of canine mammary carcinomas. This process is associated with type III collagen remodeling and tumor invasiveness, linking ECM degradation with tumor progression (13). Moreover, VCAN interacts with signalling pathways such as EGFR, HER2, and CD44, suggesting a bridge between ECM remodeling and epithelial signaling cascades with potential implications for immune modulation (14).

Importantly, the tumor ECM actively shapes immune responses in both human and veterinary oncology. In canine and feline mammary tumors, collagen characteristics quantified by second harmonic generation (SHG) imaging—such as fiber length, width, straightness, and boundary integrity—serve as strong prognostic markers. Specifically, in canine mammary carcinomas, denser, longer, and straighter intratumoral collagen fibers correlate with poorer overall survival. Similar collagen features are observed in feline mammary tumors and human breast cancer, highlighting translational relevance across species (6, 7). These collagen signatures correlate with aggressive tumor biology across species and closely mirror findings in human breast cancer. Mechanistically, tumor-associated collagens modulate immunity by restricting T cell trafficking, altering macrophage phenotypes, and dampening effect or functions. This explains why dense and aligned collagen matrices often correspond to immune exclusion and poor clinical outcomes (6, 7, 15–17).

In summary, the ECM, both in human and in animals, is not merely a structural scaffold but a dynamic regulator of immune cell function, contributing to both immune suppression and activation depending on its composition, remodeling, and interactions with immune receptors (9).

### 2.1 Role of CAFs in tumor immune modulation

Within the TME, cancer-associated fibroblasts (CAFs) are key producers of the ECM and soluble factors that influence innate immunity, fostering an immunosuppressive milieu (9). CAF-derived cytokines such as IL-6, GM-CSF, and IL-8 promote monocyte differentiation into pro-tumoral M2 macrophages, which inhibit NK cell activity and support metastasis formation (18, 19).

Tumor-secreted colony-stimulating factor 1 (CSF-1) suppresses granulocytic chemokine production by CAFs, thereby limiting the recruitment of antitumor immune cells. While CSF-1R inhibition can reduce TAMs, it may inadvertently increase immunosuppressive polymorphonuclear myeloid-derived suppressor cells (PMN-MDSCs). Combining CSF-1R and CXCR2 (C-X-C Motif Chemokine Receptor 2) inhibition, which targets PMN-MDSCs migration, has been shown to improve therapeutic outcomes (20, 21).

In human PDAC, distinct CAF subsets have been identified, including inflammatory fibroblasts, myofibroblasts, and antigen-presenting CAFs capable of modulating T cell responses (22). CAFs also facilitate tumor angiogenesis by secreting VEGF, FGF-2, and remodeling the ECM. Notably, ECM degradation can release anti-angiogenic factors, demonstrating the complex regulatory role of CAFs in vascular dynamics (4).

A significant role of CAFs in carcinomas has been demonstrated for the first time in dogs. In this species, it has been shown that CAFs can induce T cell chemotaxis via the C-X-C Motif Chemokine Ligand 12-C-X-C Motif Chemokine Receptor 4 (CXCL12/CXCR4) axis. These molecules are expressed in the tumor stroma and lymphocytes, respectively, and their secretion is regulated by increased expression of TGF-*β*1 derived from CAFs, underscoring the role of these cells in modulating T cell immunity within the TME (23).

## 3 The major immune components that orchestrate the TME

### 3.1 Tumor-infiltrating lymphocytes

Within the adaptive immune response observed in the TME, tumor-infiltrating lymphocytes (TILs) represent key cellular components. Depending on their localization, they can be found either dispersed throughout the tumor stroma (sTILs) or in direct contact with malignant cells (iTILs) (24). TILs are a heterogeneous group of immune cells that play a crucial role within the tumor immune microenvironment. They include all mononuclear leukocytes (such as T lymphocytes, B lymphocytes, NK cells, and plasma cells), but exclude polymorphonuclear leukocytes (neutrophils, eosinophils, and basophils) (25). TILs consist of various population, including cytotoxic CD8^+^ T cells, various CD4^+^ T cell subsets such as Th1, Th2, Th17, regulatory T cells (Tregs), and follicular helper T cells (Tfh), as well as B cells. Each category contributes differently to the immune response, with some promoting antitumor activity (CD8^+^, Th1) and others potentially suppressing it (Tregs, Th2) (25).

Tumor-infiltrating B lymphocytes (CD20^+^) play a dual role in cancer. On one hand, they exert antitumor functions by producing antibodies, releasing pro-immunogenic cytokines and chemokines, activating the complement system, presenting antigens to T cells, and contributing to the formation of tertiary lymphoid structures (TLS) (26). On the other hand, B cells, can also promote tumor progression by secreting anti-inflammatory and pro-angiogenic factors, forming immune complexes, and enhancing complement activation. These activities foster a pro-tumorigenic environment marked by chronic inflammation and immunosuppression, which facilitates immune evasion by cancer cells (4, 27, 28).

Among the immune cells involved in shaping the tumor microenvironment, T lymphocytes play a pivotal role. CD8^+^ T cells are key players in antitumor immunity, capable of inducing apoptosis in cancer cells via cytotoxic molecules or Fas–FasL interactions. However, within tumors, they often become dysfunctional (24). CD4^+^ T helper cells have a dual role: Th1 cells support antitumor responses and can directly kill tumor cells through cytokine release (IFN-*γ* and TNF-*α*), while Th2 cells promote tumor progression by secreting anti-inflammatory mediators, such as IL-4 and IL-13, that suppress immune activity (24, 29).

In addition to CD8^+^ and CD4^+^ effector T cells, regulatory T cells play a crucial role in modulating the immune landscape of the TME. Tregs are a subset of CD4^+^ T lymphocytes that suppress immune responses, allowing tumors to evade immune control. They infiltrate the TME via specific chemokine gradients and act by releasing inhibitory cytokines (such as IL-10 and TGF-*β*), blocking antigen-presenting cells through CTLA-4, consuming IL-2, and disrupting local metabolism. They can also directly kill effector T cells, thereby promoting immunosuppression and tumor progression (24, 30, 31).

In canine mammary carcinoma, a standardized method for assessing TILs, adapted from the human International TILs Working Group, has been validated. Both stromal TILs (sTILs) and those at the invasive front increase with tumor grade, while the presence of FOXP3^+^ regulatory T cells correlate with higher malignancy. These features should be systematically incorporated into veterinary TILs evaluation and considered for stratification in clinical trials (32, 33).

### 3.2 Immune checkpoints

Immune checkpoints are physiological pathways of the immune system that are essential for modulating the immune response against pathogens and for maintaining self-tolerance in peripheral tissues. They are divided into two groups: co-stimulatory checkpoint molecules and co-inhibitory checkpoints. The latter category includes PD-1 and CTLA-4, which are the most studied in cancers and are mentioned further below.

#### 3.2.1 CTLA-4

**Figure 1:**
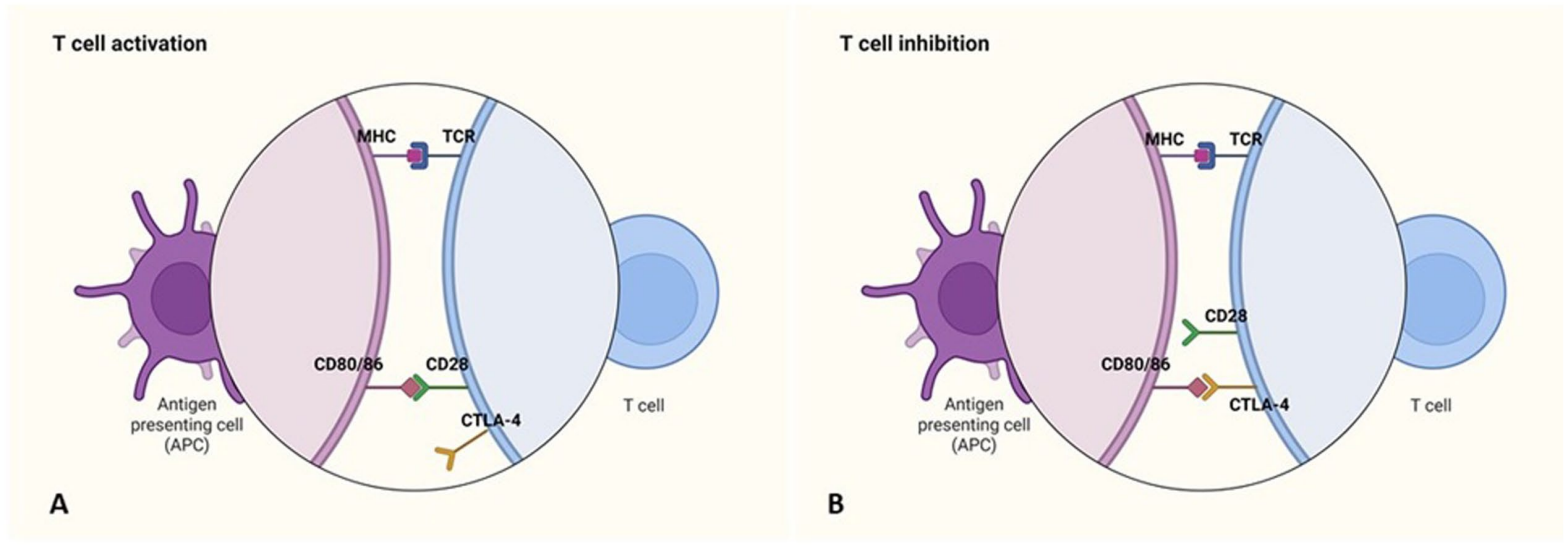
Immune checkpoint inhibitors: CTLA-4. **(A)** The activation of antigen-specific T cells requires costimulatory signals, which are generated through the recognition of antigens presented by MHC molecules on antigen-presenting cells (APCs), along with the binding of the T cell surface molecule CD28 to its ligands (CD80/86) on APCs. **(B)** The expression of the inhibitory receptor CTLA-4 on T cells leads to their inhibition. Like CD28, CTLA-4 binds to CD80/86 on APCs, but instead of promoting activation, it blocks the costimulatory signals necessary for T cell activation. Created in https://BioRender.com.

Immune checkpoints play a key role in regulating effector T cell activation through distinct, non-redundant mechanisms. CTLA-4 (Cytotoxic T-Lymphocyte Antigen-4) is one of the first identified checkpoints and modulates the early phases of T cell activation by competing with CD28 for binding to CD80/86 on antigen-presenting cells. Due to its higher binding affinity, CTLA-4 inhibits costimulatory signaling, thereby limiting T cell activation (Figure 1). It is primarily expressed on Tregs but can also be found in activated effector T cells. This pathway contributes to immune tolerance and prevents autoimmunity (34, 35).

Preclinical studies based in murine models of melanoma, have shown that CTLA-4 blockade can enhance antitumor immunity by reducing Treg-mediated suppression and restoring effector T cell function (36).

#### 3.2.2 PD-1 and PD-L1/PD-L2

**Figure 2:**
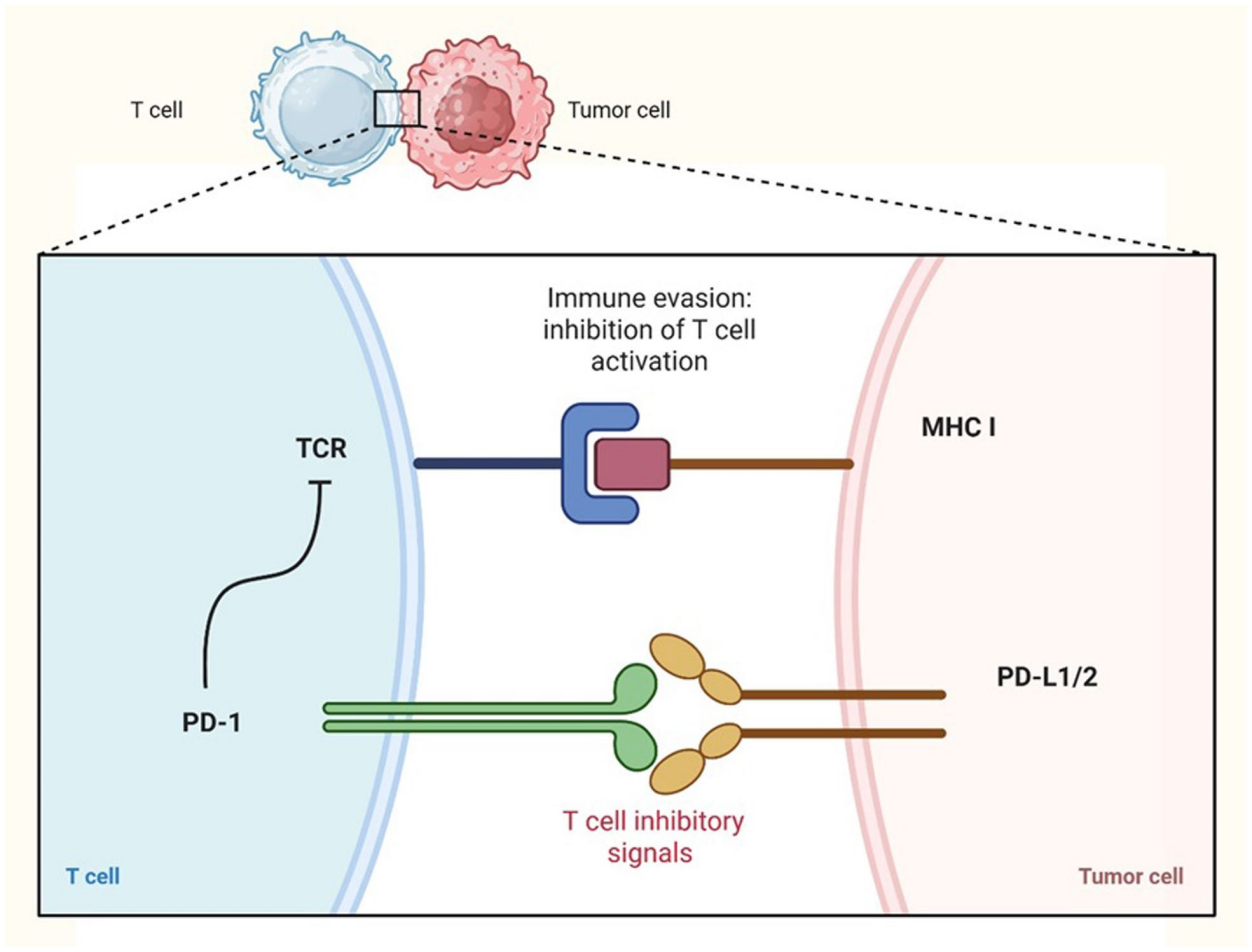
Immune checkpoint inhibitors PD-1: immune evasion by tumor cells. Tumor cells can evade the immune response by upregulating the expression of PD-L1 or PD-L2 on their surface. These ligands bind to the PD-1 receptor on T cells, leading to the inhibition of T cell activation and allowing tumor cells to escape immune surveillance. Created in https://BioRender.com.

**Figure 3:**
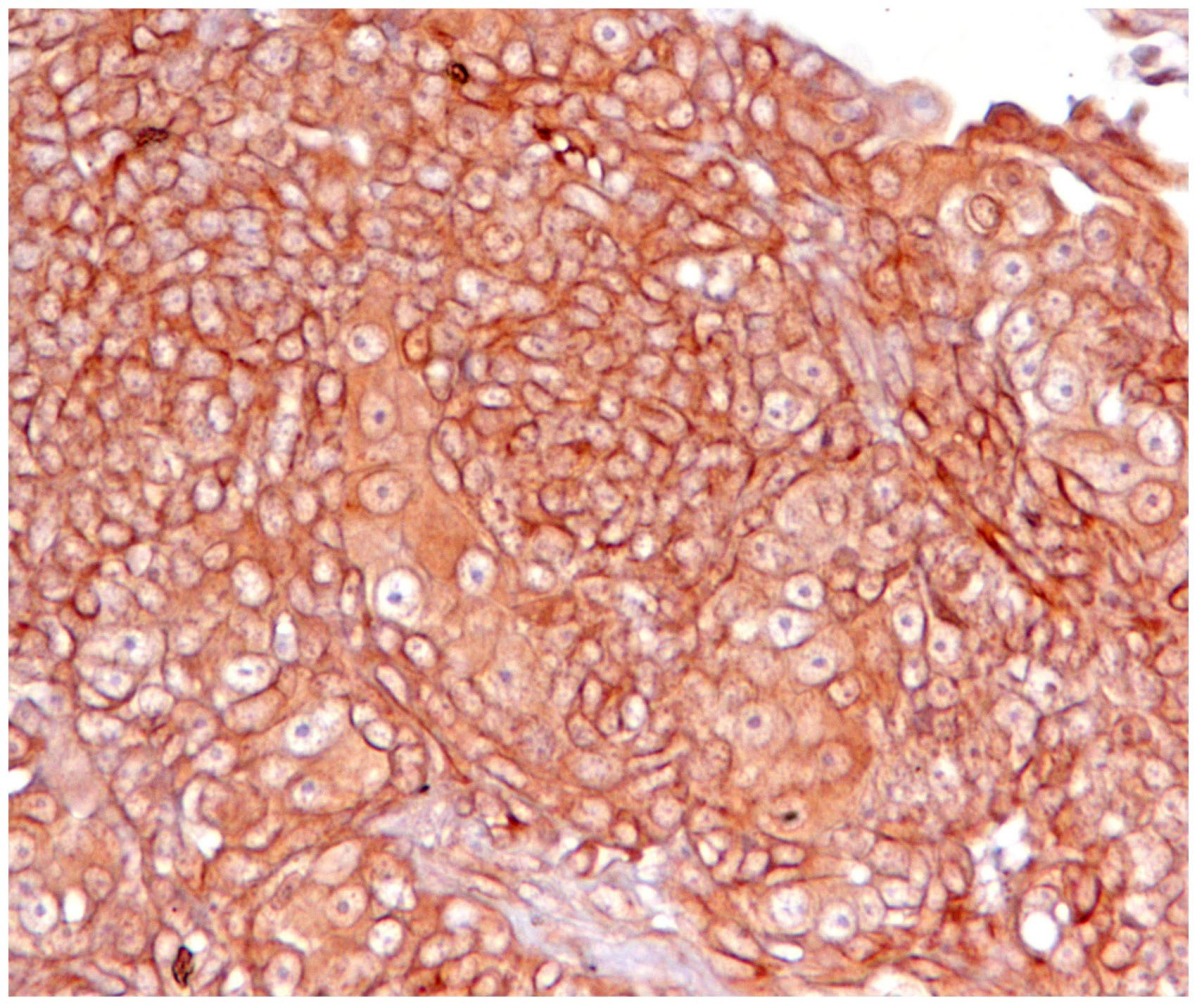
Canine squamous cell carcinoma, immunohistochemistry for PD-L1: neoplastic cells show strong positivity with membrane labelling. Cytoplasmic staining is also present but is not considered specific. Technical specifications: PD-L1/CD274 Rabbit pAb, ABClonal, A1645, with positive control (not shown) using normal canine placenta.

The PD-1 (Programmed cell death protein 1) immune checkpoint plays a crucial inhibitory role in T cell function within the TME. Upon antigen stimulation, PD-1 is expressed on T cells, B cells, and myeloid cells, while its ligands PD-L1 and PD-L2 are typically expressed by tumor cells (Figures 2, 3) and dendritic cells (DCs). Unlike CTLA-4, which inhibits T cell activation at the priming phase, PD-1 suppresses T cell activity through interactions with its ligands within the TME (35). In human pancreatic ductal adenocarcinoma tumor-infiltrating γδ T cells expressing PD-L1 suppress cytotoxic T cell and Th1 responses via PD-1 engagement, contributing to immune evasion (35). However, the immunosuppressive effect of PD-L1 may vary depending on the cell type; in murine models, PD-L1 expression by NK cells inhibited DCs activation without directly affecting effector T cells (35, 37).

Mechanistically, PD-1 engagement interferes with key signaling pathways such as Ras and PI3K, impairing T cell proliferation and metabolism (mitochondrial respiration and glycolysis) (38). PD-1/PD-L1 interactions also promote Treg induction, especially when PD-L1 is expressed by DCs. Checkpoint blockade in murine cancer models reduces Treg infiltration and enhances CD8^+^ T cell IFN-*γ* production (39).

In human non-small cell lung cancer, particularly epidermal growth factor receptor (EGFR)-mutant subtypes, PD-1/PD-L1 inhibitors show limited efficacy. This is partly due to tumor expression of immunoglobulin-like transcript-4 (ILT4), an immunosuppressive molecule upregulated by mutant EGFR via AKT and ERK1/2 pathways (40).

The study of immune checkpoints is of growing and current interest in veterinary oncology (41–44). Incorporating validated antibody clones and harmonized scoring systems will be crucial to ensure comparability across studies.

### 3.3 Tumor-associated macrophages

TAMs are the most abundant immune cells within the TME and are key mediators of chronic inflammation in solid tumors. Activated macrophages release Reactive Oxygen Species (ROS), Reactive Nitrogen Species (RNS), TNF-*α*, IL-6, IL-12, and IL-1β, contributing to a pro-tumorigenic environment. Upon IFN-*γ* and Toll-Like Receptor (TLR) ligand stimulation, they can exert cytotoxic effects via nitric oxide production (9).

**Figure 4:**
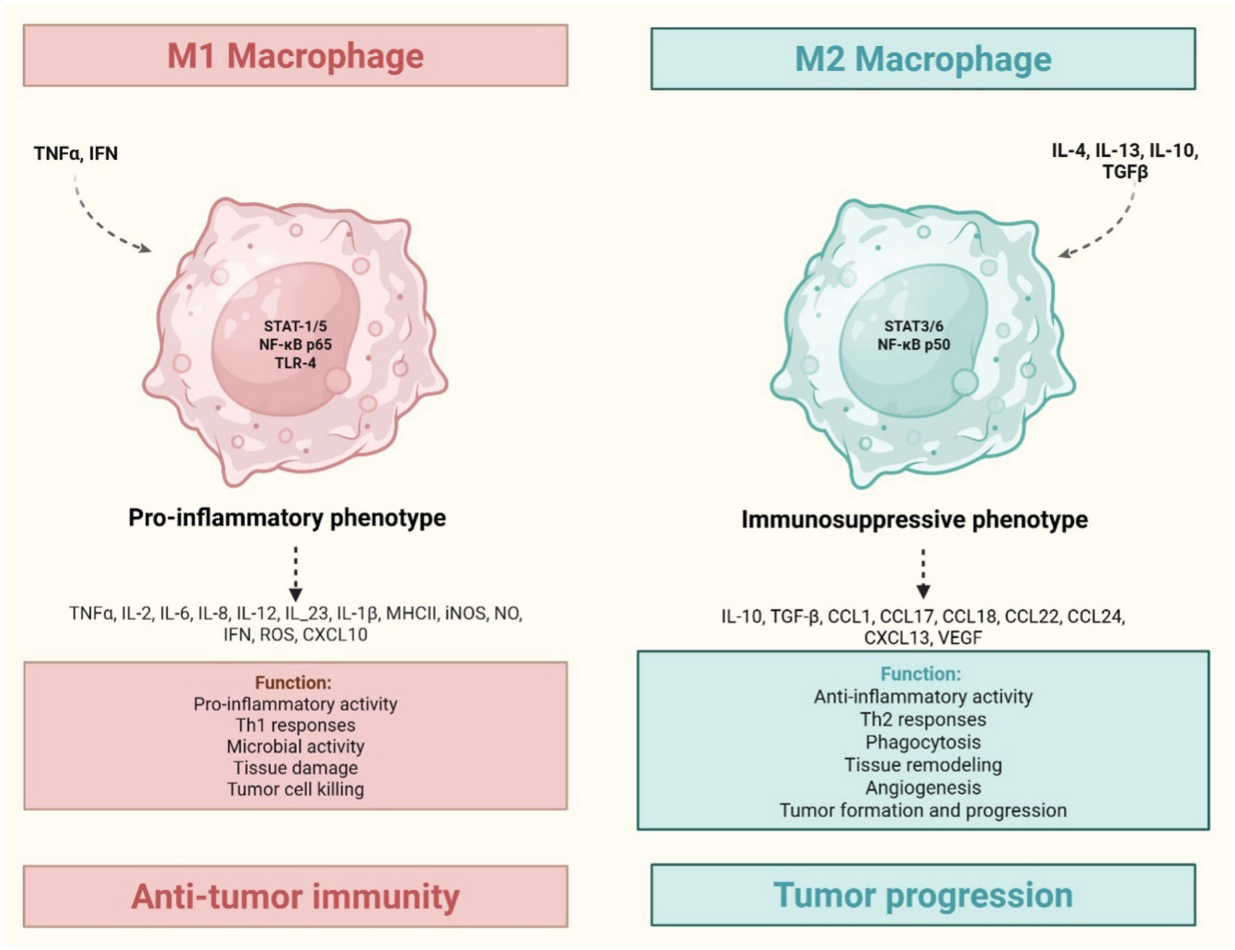
Tumor-associated macrophages: M1 (inflammatory) and M2 (anti-inflammatory) phenotypes. TNF and IFNγ induce M1 macrophages polarization, leading to a pro-inflammatory phenotype. This is associated with Th1 response and the release of Reactive Oxygen Species (ROS), Reactive Nitrogen Species (RNS), TNF-*α*, IL-6, IL-12, and IL-1β, all contributing to a pro-tumorigenic environment. Conversely, IL-4, IL-13, IL-10, TGF-β induce M2 macrophages polarization, resulting in an immunosuppressive phenotype. This leads to anti-inflammatory activity, extracellular matrix remodeling (e.g., ADAMTS 15/versikine axis) that fosters invasion, and suppression of anti-tumor immunity, primally mediated by IL-10 and TGF-β. Created in https://BioRender.com.

Tumor-derived signals promote macrophage polarization toward a pro-tumoral M2-like phenotype, supported by IL-4, IL-13, IL-10, TGF-*β*, hypoxia, immune complexes and tumor metabolites. While M1 macrophages exhibit anti-tumor properties, M2 macrophages facilitate tumor growth and progression (Figure 4) (3, 45). TAMs are heterogeneous and plastic, evolving during tumor development, they promote angiogenesis, particularly through Tie2^+^ monocyte-derived cells that secrete VEGF (46).

In breast cancer, perivascular TAMs enhance metastasis by aiding tumor cell intravasation (47).

TAMs also suppress anti-tumor immunity via IL-10 and TGF-*β*, remodel the ECM through matrix metalloproteinases, and induce epithelial-mesenchymal transition, fostering invasion (9). At metastatic sites, TAMs support tumor cell survival, e.g., through interactions between vascular cell adhesion molecule-1 and its ligand α4 integrin in pulmonary metastasis (48). Despite M2-like features, TAMs retain functional plasticity and can be reprogrammed toward an M1-like phenotype with anti-tumor potential (3, 9).

TAMs frequently co localize with remodeled collagen and can either enforce or relieve matrix imposed immune exclusion. Recent comparative work integrates TAM phenotypes with collagen architecture in canine and human mammary carcinomas, providing a matrix aware framework for TAM targeted therapy and for combining macrophage modulating strategies with stroma directed interventions (8, 49).

### 3.4 Innate and innate-like immune cells in the TME

In addition to conventional T lymphocytes and TAMs, several other immune cell populations play key roles in shaping the TME. Among these, NK cells, NKT cells, and unconventional T cells, such as γδ T cells, are critical components of the innate and innate-like immune response. These cells contribute to tumor immunosurveillance and antitumor immunity but can also support tumor progression depending on the local signals within the TME. The following is a concise overview of the mechanisms by which these immune cells influence the TME and tumor development.

NK cells are key innate lymphoid cells that mediate antitumor activity through cytotoxic granule release and death receptor engagement. They recognize cells with reduced MHC-I via activating receptors such as NKG2D and NKp46 (50–52). Tumors can evade NK cells by downregulating ligands or upregulating inhibitory signals (53). NK cells also shape the TME by recruiting dendritic cells via chemokines—a process disrupted by PGE2 (54, 55). Their function is regulated by cytokines like IL-15 and IL-1R8 (3, 9, 56, 57).

NKT cells bridge innate and adaptive immunity, expressing both NK markers and TCRs. Type I (iNKT) cells exert antitumor effects by activating dendritic and T cells, while Type II NKT cells are associated with immunosuppression and tumor promotion (58–60). Their impact is mediated by cytokine secretion (IL-12, IL-21, IL-2) or suppression (3, 9).

*γ*δ T cells are unconventional T lymphocytes that recognize stress-induced ligands independently of MHC. They contribute to tumor control via direct cytotoxicity (NKG2D, DNAM-1, TRAIL) and cytokine production (IFN-γ, TNF-*α*) (61–63). However, in certain TMEs, they may acquire a γδT17 phenotype, secreting IL-17 and IL-1β, which promotes angiogenesis and immune evasion (3, 9, 64, 65).

Other important components beyond NK, NKT, and unconventional T cells in the TME are Myeloid-Derived Suppressor Cells (MDSCs). MDSCs are immature myeloid cells divided into monocytic (M-MDSCs) and granulocytic (G-MDSCs) subsets, both able to suppress T cell activity and promote tumor progression through immunosuppression and angiogenesis (9, 66). Their expansion and recruitment in tumors are driven by cytokines like GM-CSF, IL-6, and VEGF, which activate STAT3 signaling to maintain their immature, suppressive phenotype (3, 67). M-MDSCs are more prevalent in tumors and can differentiate into TAMs, influenced by hypoxia and HIF-1α (9) M-MDSCs inhibit T cell proliferation via secretion of suppressive factors such as L-arginine, iNOS, TGF-*β*, IL-10, and IDO, causing nutrient depletion and accumulation of toxic metabolites in the TME (20). They also impair T cell function through nitric oxide production and transfer of methylglyoxal, leading to immune dysfunction (68, 69) Moreover, M-DSCs promote metastasis and angiogenesis by secreting IL-6, Bv8, VEGF, and MMP-9, which facilitate tumor growth and vascularization (3, 70). Their presence correlates with worse outcomes and resistance to immunotherapy, being them important but challenging therapeutic targets (9).

## 4 Immune landscape of the tumor microenvironment in canine and feline spontaneous neoplasms

### 4.1 Canine and feline mammary carcinoma

Mammary carcinoma is one of the most frequent neoplasms in both dogs and cats, with distinct biological behavior between species. In dogs, around 55% of mammary tumors are malignant, but often less aggressive than in cats. Late or absent spaying is the main risk factor (71). In cats, Feline Mammary Carcinoma (FMC) is typically highly malignant, with a strong tendency for invasion and metastasis. The risk is significantly reduced by early spaying, while progestin contraceptives increase susceptibility (24, 72). FMC is recognized as a highly comparable spontaneous model of human breast cancer due to its metastatic pattern (regional lymph nodes and lungs), as well as its clinical and histopathological features (6).

Among the various subtypes, FMC shares strong similarities with the basal-like subtype of human breast cancer, characterized by the lack of ER, PR, and HER2 expression, and positivity for basal cytokeratins (73, 74). In humans, this subtype is linked to a highly immunosuppressive TME (6). In cats, Tregs infiltration in basal-like and luminal FMCs has been associated with shorter disease-free interval (DFI) and tumor specific survival (TSS), defining an “immunosuppressed” subgroup within the basal-like phenotype (74).

Peripheral blood leukocyte counts, and neutrophil-to-lymphocyte ratio (NLR) have emerged as prognostic indicators. Higher NLR values were associated with shorter DFI and TSS, highlighting its potential as a preoperative prognostic biomarker and therapeutic guide (75–77).

In addition, high serum levels of VEGF-*α*, VEGFR-1/2, and PD-1/PD-L1 have been observed in aggressive FMC subtypes such as HER2^+^ and triple-negative tumors, and are associated with increased TILs (6, 78).

**Figure 5:**
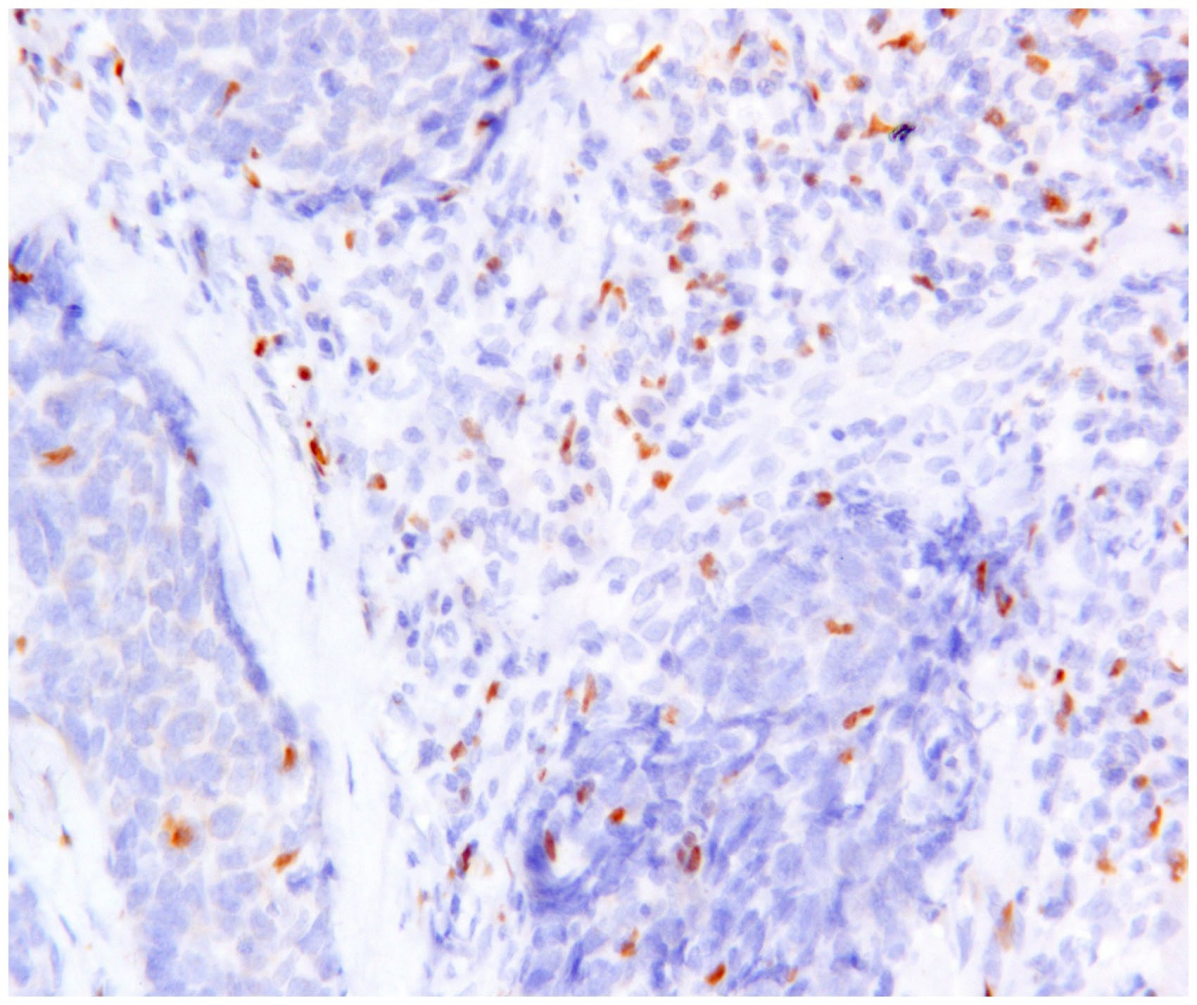
Canine mammary carcinoma, immunohistochemistry for FoxP3: numerous FoxP3^+^ regulatory T lymphocytes with nuclear immunoexpression are present in supporting stroma and in the neoplastic tissue.

TILs play a critical role in the TME of canine mammary carcinoma (CMC), where Tregs (Figure 5) contribute to the suppression of anti-tumor immune responses. Their interaction with other immunosuppressive cells—such as Th2 cells, M2-polarized macrophages, and myeloid-derived suppressor cells (MDSCs) —further facilitates tumor progression (25).

While high levels of TILs in human breast cancer are generally associated with a better prognosis, in CMC, elevated TILs in the stromal compartment are linked to poorer outcomes. Notably, Tregs increase proportionally with TILs density, contributing to the formation of an immunosuppressive barrier at the invasive front of the tumor (32, 79). In addition to stromal TIL density, the organization of TILs into tertiary lymphoid structures (TLSs) has been described in dogs and is observed in high-grade tumors, further supporting the association of TILs and TLSs with an aggressive tumor phenotype (80).

Studies have shown that dogs affected by triple-negative CMC with marked inflammatory infiltrates have poorer survival. Increased levels of CD3^+^, CD4^+^ T cells, as well as TAMs have been identified as potential prognostic indicators in these cases (81). Additionally, Tregs infiltration is linked to increased malignancy, metastasis, and higher histological grade (32, 82).

TAMs contribute to tumor aggressiveness and have been associated with larger tumor size, lymphatic invasion, and increased Ki67 expression (83). The macrophages polarization, specifically toward the M2 subtype, plays a critical role in the progression of CMC. Tumors with a predominance of M2 polarized (CD204^+^) TAMs were associated with significantly shorter tumor-specific median survival and were more frequently observed in aggressive tumor phenotypes. In contrast, tumors with a higher proportion of IBA1^+^ cells were associated with a more favorable prognosis. These findings suggest that the TAMs polarization toward the M2 phenotype may have a detrimental impact on disease outcome (84). CD204^+^ macrophages infiltration is also more prominent in HER2-overexpressing and triple-negative subtypes compared to luminal types, suggesting a potential role in promoting tumor aggressiveness (85).

Suppressor of cytokine signaling proteins (SOCS1 and SOCS3) are key regulators of immune responses. SOCS1 expression in macrophages is associated with a more aggressive tumor phenotype, while SOCS3 correlates with an anti-tumor response. In CMC, SOCS3 expression in macrophages is associated with lower metastasis, whereas SOCS1 correlates with worse outcomes (86, 87).

PD-L1 expression has been investigated in canine mammary carcinoma, with significant discrepancies among studies. The reported prevalence of expression ranges from 3% to as high as 80–100% in the literature. This divergence is attributed to methodological and analytical differences among studies, as well as the lack of standardized evaluation guidelines, unlike in humans, where membrane expression is considered mandatory (41–43).

### 4.2 Canine colorectal carcinomas

Colorectal cancer (CRC) is one of the most common malignancies in humans and a leading cause of cancer-related mortality, with chronic inflammation playing a key role in its pathogenesis. In veterinary medicine, dogs are the species most frequently affected by spontaneous colorectal tumors, making them a valuable comparative model for human CRC studies (88, 89). In both humans and dogs, colorectal carcinoma is often associated with a poor prognosis, due to high rates of local recurrence in dogs and distant metastases in humans (90, 91).

Therefore, inflammation plays a significant role in the development of CRC, influencing the progression from adenoma to adenocarcinoma. The immune microenvironment, particularly the interaction between tumor cells and immune cells, affects this transition. Macrophages are especially involved, secreting pro-inflammatory cytokines that promote malignancy (92, 93). In both humans and dogs, adenomas that progress to CRC are characterized by a high density of mast cells, which secrete pro-angiogenic and pro-inflammatory factors that contribute to tumor development (94, 95).

In humans, a higher infiltration of CD3^+^ T lymphocytes in tumors correlates with better survival outcomes, while lower levels are associated with poorer prognosis. Conversely, TAMs are linked to a more aggressive phenotype (96, 97).

In dogs with colon adenocarcinoma, however, TILs infiltration is lower compared to humans, and the roles of specific T-cell subpopulations remain unclear. TAMs were found to be more abundant in adenocarcinomas than in adenomas, suggesting a potential link to malignancy (98). However, further research is needed to clarify their precise role in tumor progression because another study reported the opposite trend, observing a higher infiltration of TAMs in adenomas compared to adenocarcinomas (95).

Ki67, a marker of cell proliferation, has been controversial role as a prognostic indicator in human CRC (99–101). In dogs, Ki67 expression, along with TAMs infiltration and mast cell presence, has been associated with CRC malignancy. Specifically, Ki67 correlates with higher mitotic indices, larger tumor size, necrosis, and vascular invasion. Additionally, mast cells appear to serve as indicators of poor prognosis in canine CRC (95).

### 4.3 Canine visceral hemangiosarcoma

Canine visceral hemangiosarcoma (HSA) is a relatively common, highly malignant tumor originating from vascular endothelial cells. It frequently affects highly vascularized visceral organs such as the spleen, liver, heart, and skin. Clinical signs often appear suddenly due to tumor rupture, which commonly causes haemorrhagic effusions in the peritoneal and pericardial cavities. HSA is characterized by early and widespread metastasis, leading to a poor prognosis with an average survival time of 4 to 8 weeks despite radical surgical treatment (102, 103).

The previously discussed M1/M2 macrophage polarization appears to play a significant role in HSA. Kerboeuf et al. (102) employed CD206 as a specific marker for M2 macrophages and used CD204 to label the overall macrophage population. This approach contrasts with prior veterinary literature (85, 104), where CD204 was often used as an M2-specific marker. In this study, a higher number of total macrophages, M2 macrophages, and an increased M2-to-total macrophage ratio were observed within tumor hotspots and in the surrounding neoplastic tissue. In contrast, non-tumoral regions predominantly contained CD206^−^ macrophage populations.

Further studies have confirmed that canine splenic HSA is highly immunogenic. An accumulation of FoxP3^+^ immune cells (Tregs), potentially acting through the CTLA-4 immune checkpoint, appears to contribute to immunosuppression, tumor progression, and metastasis (105).

Elevated levels of CD20^+^ B cells were significantly associated with increased metastatic risk, in line with observations in both canine oral melanoma and human oral squamous cell carcinoma (106, 107). Macrophages were identified using the pan-histiocytic marker Iba-1, which revealed a correlation between Iba-1^+^ cell number and clinical tumor stage; however, no significant prognostic relevance was observed (105).

### 4.4 Canine soft tissue sarcomas

Canine soft tissue sarcomas (STSs) are a heterogeneous group of mesenchymal tumors, accounting for approximately 15% of all cutaneous and subcutaneous neoplasms. These tumors share common features including challenging surgical removal, a high risk of local recurrence, and systemic metastases in about 30% of cases (108).

STSs have long been considered immunologically inactive or “cold” tumors. However, especially in human medicine, recent findings have highlighted the role of TILs, TAMs and the expression of immune checkpoint molecules such as PD-1, PD-L1, and PD-L2 in modulating tumor behavior and activating the immune response. High PD-L1 expression has been linked to poor prognosis, while increased M2 macrophages are associated with treatment resistance and worse outcomes. In contrast, M1 macrophages and CD8^+^ T cells are linked to more favorable clinical outcomes (109–111).

**Figure 6:**
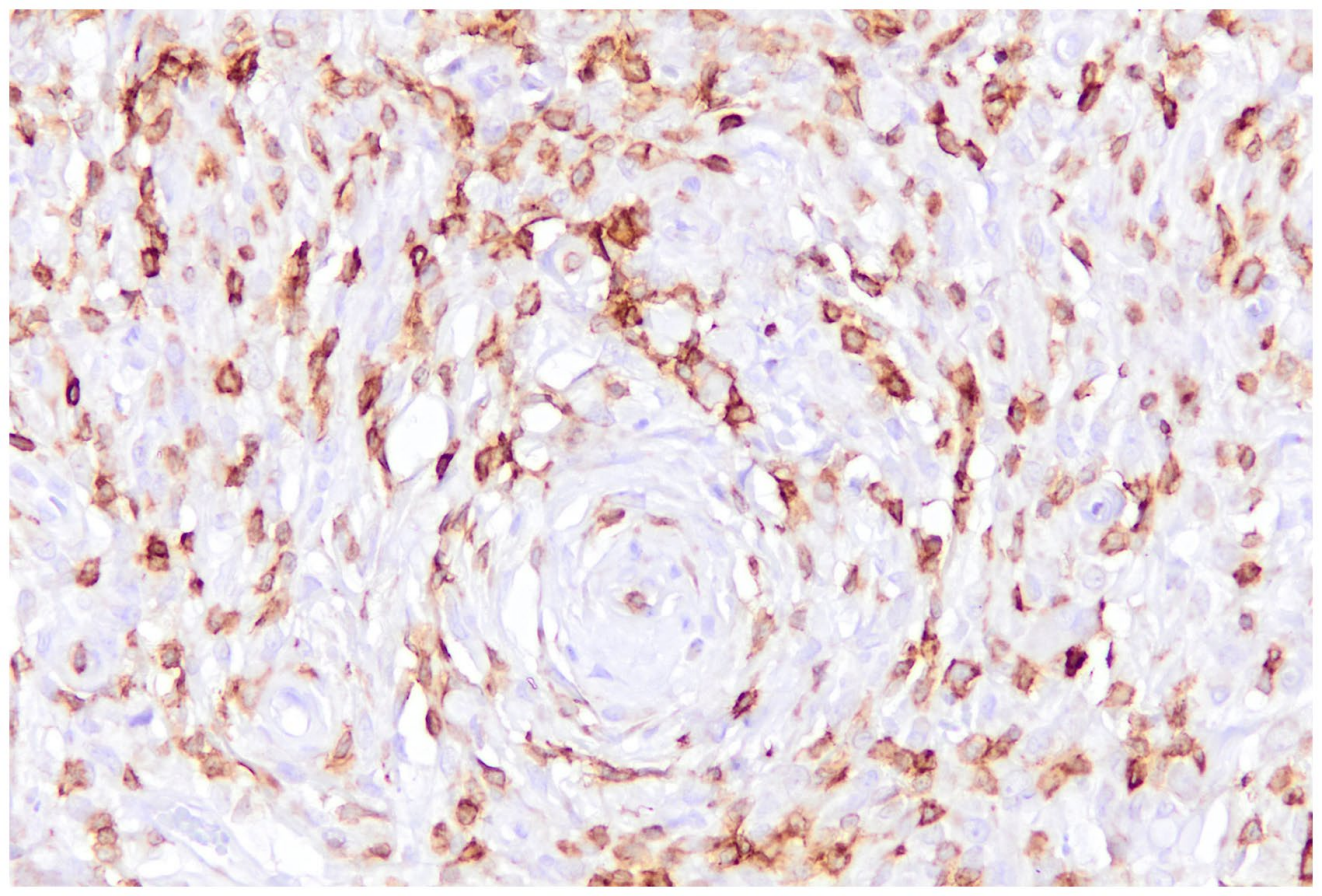
Canine perivascular tumor, immunohistochemistry for CD3: numerous tumor-infiltrating lymphocytes with a T-cell immunophenotype are intermingled with the sarcomatous cells.

In canine STSs, characterization of the TME is still limited but growing. Variations in TILs density and composition have been observed across different sarcoma histotypes. For example, myxosarcomas exhibit high infiltration of B lymphocytes, which is associated with an increased presence of Tregs, suggesting a potentially immunosuppressive TME that may be linked to a worse prognosis. In perivascular wall tumors, both B and T (Figure 6) lymphocytes are present in high numbers, whereas Tregs are less represented. Leiomyosarcomas, liposarcomas, and fibrosarcomas tend to show low TILs infiltration, but Tregs density increases with histological grade in leiomyosarcomas and fibrosarcomas (4, 26, 112).

TAMs in canine STSs have been studied using the immunohistochemical marker Iba-1. These cells were investigated in relation to mitotic activity, differentiation, and necrosis. Among these parameters, only mitotic activity showed a significant association with high TAMs infiltration. The lack of distinction between M1 and M2 macrophages represents a limitation, as increased mitotic rates could reflect M2 polarization, which is generally associated with tumor progression and poor prognosis (113).

The expression of immune checkpoints such as PD-1, PD-L1, and PD-L2 in STSs may further influence tumor progression. In a study assessing all three histological grades of canine STSs using the Dennis grading system, PD-L1 expression increased with tumor grade, and PD-1/PD-L2 expression was especially associated with poorly differentiated (grade 3) tumors. These markers may thus have prognostic relevance, as shown in human STSs, where PD-L1 overexpression is linked to higher malignancy and shorter survival (111, 112, 114, 115).

### 4.5 Canine melanocytic tumors

Canine oral melanoma (OM) is a malignant tumor originating from melanocytes and exhibits a particularly aggressive biological behavior, characterized by a high risk of local recurrence and metastasis. Consequently, it is frequently associated with a poor prognosis and limited response to conventional therapies (116, 117). In contrast, among canine melanocytic tumors, the cutaneous form tends to be less aggressive (118).

Immune checkpoint molecules, including PD-1/PD-L1 and CTLA-4 play a central role in tumor immune evasion. In both oral and cutaneous melanomas in dogs, PD-L1 expression has been detected on tumor cells as well as on TILs (41, 43). Moreover, elevated CTLA-4 expression on lymphocytes correlates with a poorer prognosis (119).

A recent study used RNAscope *in situ* hybridization to investigate the expression of PD-1, PD-L1, and CTLA-4 in the TME of canine oral melanoma. PD-L1 was expressed in all tumors, mainly by neoplastic cells and TAMs, while PD-1 and CTLA-4 were predominantly expressed by CD3^+^ TILs (43). Interestingly, PD-1 gene expression in tumor cells was associated with a higher mitotic index, suggesting a possible pro-tumoral role via the mTOR pathway, as hypothesized in human melanoma (120). Moreover, PD-1 and PD-L1 mRNA levels appeared higher in melanomas (oral and cutaneous) compared to benign cutaneous melanocytomas (121).

TILs can exert both anti-tumor effect, such as those mediated by cytotoxic T lymphocytes (CTLs), and pro-tumor function, as seen with Tregs. In canine oral melanoma, higher infiltration of CD8^+^ and CD4^+^ T cells have been observed in early-stage tumors (stages I–II) and in cases with longer survival. FoxP3^+^ Tregs cells were less prevalent and not directly linked to prognosis. A marked lymphocytic infiltration, especially by CD8^+^ T cells, was associated with improved survival compared to tumors with sparse or absent infiltrates. Clinical staging and assessment of tumor aggressiveness were based on WHO-adapted criteria (122–124).

Oral melanomas in dogs show higher levels of FoxP3^+^ regulatory T cells and IDO^+^ (indoleamine 2,3-dioxygenase, an immunoregulatory enzyme implicated in suppressing T-cell) inflammatory cells compared to cutaneous melanomas and melanocytomas. FoxP3 was also expressed by neoplastic cells, potentially mimicking Treg-induced immunosuppression. IDO^+^ cells, mainly dendritic cells and macrophages, were linked to increased risk of metastasis and death. The positive correlation between Tregs and IDO^+^ cells suggest a cooperative immunosuppressive mechanism within the TME (125, 126).

TAMs are a key component of TME in canine melanocytic neoplasms. A study has shown that Iba-1 expression is higher in cutaneous melanomas than in melanocytomas. CD163, a marker commonly associated to the M2 phenotype, shows high expression in metastatic cases and in dogs with poor outcomes, paralleling observations in human melanoma. CD204 is also present but its role as a specific M2 indicator remains controversial. Some TAMs co-express Iba-1, CD163, and CD204, reflecting phenotypic and functional overlap (84, 102, 104, 127, 128).

### 4.6 Immune contexture of poorly characterized tumors

#### 4.6.1 Canine osteosarcoma

Canine osteosarcoma is the most common primary bone tumor in dogs, characterized by aggressive local growth and a high potential for early metastasis, particularly to the lungs. It predominantly affects large and giant breed dogs and is associated with a poor prognosis despite available treatment options.

Transcriptomic analyses of canine osteosarcoma have identified three main tumor TME subtypes: immune-enriched (IE), immune-enriched with extracellular matrix features (IE-ECM), and immune-depleted (ID). The IE subtype, rich in cytotoxic CD8^+^ T cells, NK cells, and macrophages, shows strong immune activity and better clinical outcomes. The IE-ECM subtype includes immune cells but is dominated by fibroblasts and extracellular matrix, creating an immunosuppressive environment. The ID subtype, the most common, lacks immune infiltration, exhibits high tumor cell proliferation, and is linked to poor prognosis and resistance to immunotherapy. TME profiles can vary between primary and metastatic tumors (129). Tregs (FoxP3^+^) are increased in pulmonary metastases compared to tumors at primary site. Conversely, higher levels of cytotoxic T cells within metastatic sites correlate with improved survival outcomes, independent of metastatic site (129, 130).

Biller et al. (131) demonstrated that dogs with osteosarcoma have a significant increase in circulating Tregs and a decrease in CD8^+^ cytotoxic T cells compared to healthy controls, resulting in a reduced CD8/Treg ratio. This imbalance correlates with shorter survival times, suggesting the CD8/Treg ratio may serve as a valuable prognostic biomarker. Additionally, dogs with osteosarcoma exhibiting higher infiltration of CD204^+^ TAMs have been associated with longer disease-free intervals (132).

Single-cell RNA sequencing of spontaneous osteosarcoma (OSA) in treatment-naïve dogs has revealed a complex and diverse array of immune and stromal cell populations within the tumor microenvironment (TME). This advanced approach elucidates the cellular composition that influences tumor progression and immune responses, offering critical insights that could inform the development of more effective immunotherapies. Furthermore, cross-species analyses highlight a strong similarity between canine and human OSA, emphasizing the value of canine OSA as a translational model for immuno-oncology research (133).

#### 4.6.2 Canine cutaneous and subcutaneous mast cell tumors

Mast cell tumors (MCTs) are the most common skin tumors in dogs, accounting for about 16–21% of all cutaneous tumors, with variable aggressive biological behavior (134, 135).

The TME in canine cutaneous mast cell tumors (ccMCTs) varies according to histologic grade. High-grade tumors display increased infiltration of macrophages (Iba1^+^) and PD-1^+^ cells, suggesting enhanced immunogenicity and a potential link to tumor aggressiveness. T lymphocytes (CD3^+^) are present in all tumors with variable density, while regulatory T cells (FoxP3^+^) remain consistently rare regardless of grade. Macrophages appear as key components of the microenvironment and promising therapeutic targets, whereas the roles of PD-1^+^ cells and Tregs require further elucidation (134).

In both cutaneous and subcutaneous MCTs, immune infiltration is a consistent feature, with Iba1^+^ TAMs predominating. These immune cells exhibit diverse morphologies: round, spindle-shaped, or stellate that may reflect different functional polarizations. A predominance of stellate/spindle-shaped TAMs correlates with early lymph node metastasis, suggesting a pro-tumoral M2 phenotype, conversely round macrophages are more common in non-metastatic tumors (less aggressive), potentially indicative of an anti-tumoral M1 phenotype (135).

TILs, including CD3^+^ T cells and CD20^+^ B cells, are variably present. Cytotoxic T cells (CD8^+^), T-helper (CD4^+^), and Tregs (FoxP3^+^) subsets have been identified, however, their distribution and prognostic significance in MCTs remain unclear. Tregs are scarce and predominantly perivascular, showing no association with sentinel lymph node metastasis, which suggests a limited immunosuppressive role in this tumor type. In closing, the immune subset seems to be influenced by tumor location, for example subcutaneous MCTs exhibiting higher levels of TILs and Tregs cells compared to cutaneous tumors (135).

#### 4.6.3 Canine oral squamous cell carcinoma

Canine Oral Squamous Cell Carcinoma (OSCC) is one of the most common oral tumors in dogs, representing about 7–15% of all oral neoplasms. It typically affects older dogs and is characterized by aggressive local invasion and moderate metastatic potential, often leading to a poor prognosis (136).

**Figure 7:**
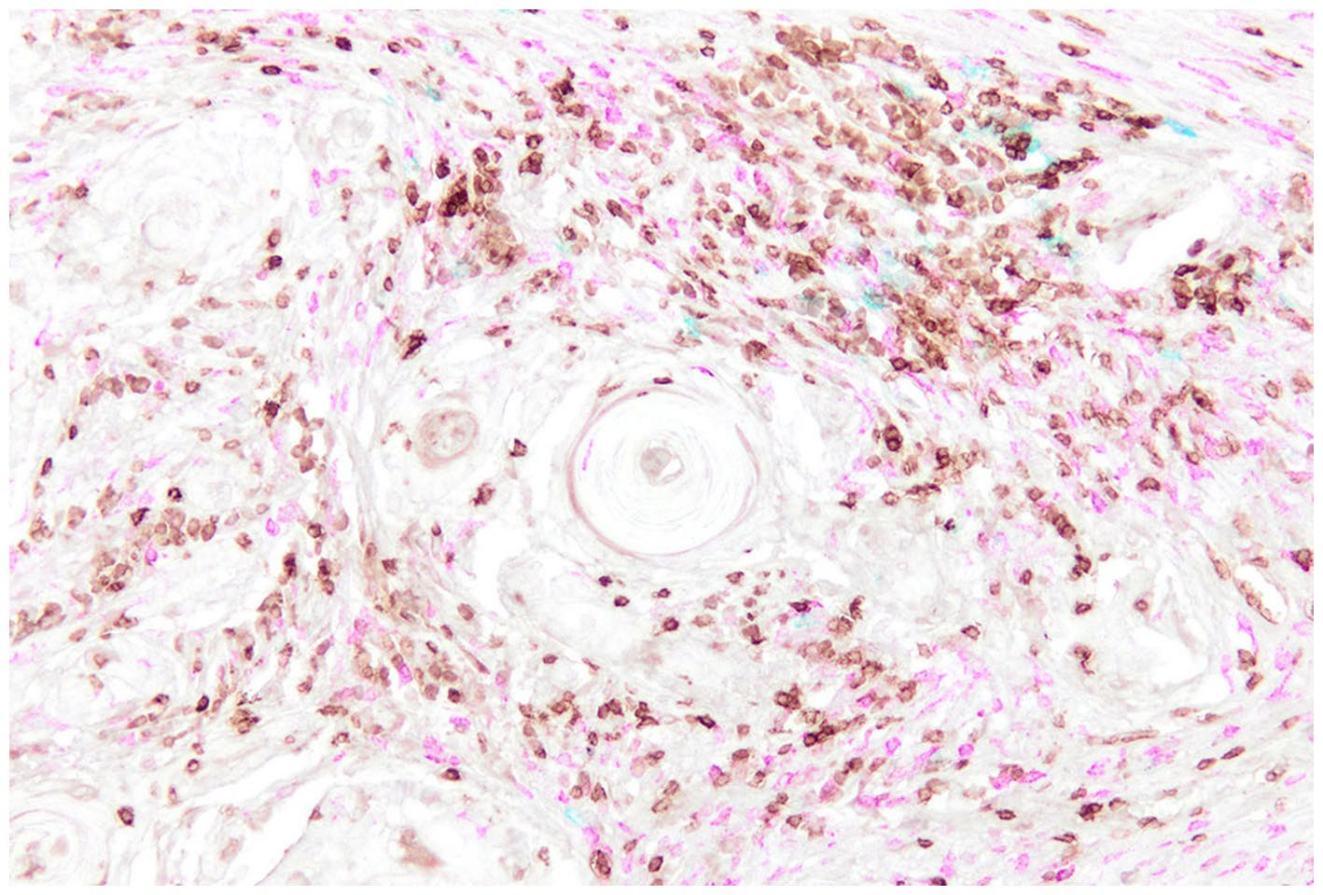
Canine Oral Squamous Cell Carcinoma, multiplex immunohistochemistry staining for CD3 (brown), CD20 (blue), and IBA1 (fuchsia) highlights the immune cell populations within the tumor microenvironment. CD3-positive T lymphocytes and IBA1-positive macrophages are the predominant infiltrating immune cells, whereas CD20-positive B cells are comparatively sparse.

Canine OSCC tumors display significant variability in immune cell infiltration within the TME, with T lymphocytes (CD3^+^) and macrophages (CD204^+^) being the predominant infiltrating populations (Figure 7). Among T cells, cytotoxic CD8^+^ T lymphocytes and NK cells were the main subsets identified. These cells were associated with increased expression of immune inhibitory checkpoints such as PD-1 and CTLA-4, markers indicative of effector cell exhaustion and immunosuppression, reflecting a highly inflamed microenvironment that also includes Tregs (136).

Furthermore, the antitumor immune response appears to be orchestrated by CD4^+^ T cells. These cells, present in the TME, show signs of activation (evidenced by increased expression of the costimulatory molecule ICOS) and their positive correlation with B cells suggests a coordinated adaptive immune response. However, CD4^+^ T cells also express inhibitory immune checkpoints such as CTLA-4, indicating functional exhaustion and reduced effector potential (137).

In addition, CD204^+^ TAMs appear to suppress antitumor immunity by promoting the recruitment of MDSCs and by producing immunosuppressive cytokines like IL-10. This immunosuppressive feedback loop is associated with more aggressive and invasive tumor behavior, likely through facilitation of epithelial–mesenchymal transition (EMT) (136, 137).

Overall, PD-1 and CTLA-4 are overexpressed in OSCC tumors with high T cell infiltration, mirroring observations in human head and neck squamous cell carcinoma (HNSCC) and represent promising immunotherapeutic targets (137).

## 5 Discussion and conclusion

The immune microenvironment is a complex and dynamic cellular network that infiltrates and surrounds the tumor. The interaction between immune cells and cancer cells profoundly influences tumor behavior in terms of progression, aggressiveness, and therapeutic response, an established concept in human oncology and an emerging area of study in veterinary medicine. Understanding the role of individual immune components within the tumor offers a promising direction for identifying new strategies to control or slow the development of spontaneous tumors in dogs and cats, mirroring efforts underway in human medicine. Beyond the immune component, in canine and feline carcinoma the ECM and CAFs play a key role in modulating tumor immunity, creating immune exclusion and poor prognosis; these findings has translational relevance as it appears to be conserved across species, supporting the integration of these components into the design of immunotherapy studies.

Nonetheless, the complexity of these interactions still poses a significant challenge to fully deciphering the mechanisms that drive carcinogenesis and immune evasion. Despite this, ongoing research continues to shed light on the opposing pro- and anti-tumor immune mechanisms governed by both innate and adaptive immunity. An additional, fundamental concept that must be considered when interpreting the TME is tumor heterogeneity, both intertumoral (differences between tumors of the same type in different individuals) and intratumoral (differences between cancer cells within the same tumor or between primary and metastatic lesions). This heterogeneity arises from genetic, epigenetic, transcriptomic, and proteomic variations and plays a critical role in shaping immune evasion, therapy resistance, and disease progression (138). The dynamic nature of these variations, in both space and time, directly impacts how tumors respond to the immune system and to therapeutic interventions, and must therefore be accounted for in future veterinary oncology research.

Innovative therapeutic approaches could involve modulating the tumor immune microenvironment, similar to strategies currently explored in human medicine, such as macrophage repolarization or the use of immune checkpoint inhibitors. In this context, immune checkpoint inhibitors targeting PD-1, PD-L1, and CTLA-4 have been investigated in the treatment of canine melanoma. However, despite these efforts, therapeutic responses in dogs remain limited. This limited efficacy may be explained by several factors, including heterogeneous methods for PD-L1 assessment, differences in tumor immunogenicity, variations in drug pharmacokinetics, and a lack of prospective biomarker-driven clinical trials (116, 139, 140). Therefore, basic research studies that thoroughly characterize the tumor immune microenvironment in canine and feline neoplasms are urgently needed to accurately select patients within a personalized medicine framework.

Moreover, the development of novel therapeutic approaches in veterinary medicine that target the specific composition of the TME, distinctly shaped by the type of neoplastic process involved, may also provide valuable insights for human medicine. This reinforces the One Health concept and strengthens the link between veterinary and human oncology. Indeed, much of the current knowledge discussed in this review stems from translational research studies, highlighting the reciprocal benefit of comparative oncology.

Future therapeutic perspectives in companion animals must rely on a thorough characterization of the immune tumor microenvironment across the main types of spontaneous neoplasms affecting dogs and cats. To this end, it is crucial to resolve current uncertainties regarding the pro- and anti-tumor roles of immune cells within specific tumor types. For example, a more precise identification of M1 and M2 macrophage subsets in the TME would help confirm the hypothesized pro-tumoral function of M2-polarized macrophages. Likewise, the expression of inhibitory immune checkpoints such as CTLA-4, PD-1/PD-L1, and PD-L2 appears to be strongly associated with regulatory T cells, which mediate immunosuppressive activity and contribute to tumor immune evasion.

In this regard, tumor types recognized as particularly aggressive in veterinary medicine, such as high-grade mammary carcinoma, oral melanoma, and visceral hemangiosarcoma, which are extensively discussed in this review show increased expression of inhibitory immune checkpoints, often associated with a higher presence of Tregs. Moreover, macrophage infiltration, likely polarized toward the M2 phenotype, appears to influence the highly malignant behavior of these neoplasms.

These findings underscore the critical importance of investigating the tumor immune microenvironment as a fundamental factor in understanding and potentially modulating tumor aggressiveness in companion animals. However, the precise impact of these immune components on prognosis, clinical presentation, and overall disease progression remains to be fully elucidated.

For greater accuracy and comparability of research data, it is essential to adopt validated immunohistochemical markers and scoring systems. To this end, the authors propose both material and analytical criteria for the evaluation of key components of the TME in veterinary medicine, as summarized in Table 1.

**Table 1:** Scoring systems and immunohistochemical panels for evaluation of tumor immune microenviroment in canine tumors.

Immune cells and immune checkpoints	Scoring system	Antibodies panel	Antibodies clones and details	Subcellular location	References
TILs	*H-E based:* TILs working group scoring system adapted in dog				(32)
	*IHC phenotype:* Not yet standardized – digital quantification suggested	CD3	CD3 (clone CD3-12, Leucoytes Antigen Laboratory, UCDavis; clone F7.2.38, mouse monoclonal, Dako)	Membrane (CD3, CD8, CD20)	CD3: (32, 112)
		CD8	CD8 (rabbit polyclonal, Abcam, ab4055)		CD8: (141)
		CD20	CD20 (rabbit polyclonal, Invitrogen)		CD20: (112)
		FOXP3	FOXP3 (rat monoclonal FJK-16 s, Thermofisher)	Nuclear (FOXP3)	FOXP3: (32, 126)
TAMs	*IHC phenotype:* Not yet standardized – digital quantification suggested	Iba1	Iba1 (goat polyclonal Novus; clone MABN92, Merck Millipore)	Cytoplasm-membrane (IBA1)	Iba1: (104)
		CD204	CD204 (clone SRA-E5, mouse monoclonal Abnova)	Membrane (CD204, CD206, CD163)	CD204, CD206: (102)
		CD206	CD206 (rabbit polyclonal, Abcam)		
		CD163	CD163 (clone EDHu-1, Bio-Rad)		CD163: (104)
PD-L1	TPS (Tumor Proportion Score), CPS (Combined Positive Score)	PD-L1	PD-L1/CD274 Rabbit pAb, ABClonal, A1645	Membrane	PD-L1: (41)

The grading system developed by the TILs Working Group in human oncology, when applied to canine mammary carcinomas (32), has proven to be a robust and reproducible method across species. A correlation has been observed between increased TIL density and higher histological grade. However, further prognostic studies are needed to stratify affected populations and to assess whether TIL scoring can serve as an independent prognostic marker. For this reason, a consensus scoring system is needed to ensure consistency across studies, and the authors advocate for its adoption in future research on this topic.

The use of validated and cross-reactive antibody clones is also essential to ensure analytical comparability and reproducibility of results. Furthermore, analytical concordance is crucial—for instance, the mandatory identification of membranous-specific staining for PD-L1 is required to avoid false-positive results when evaluating this immune checkpoint.

Accurate cellular quantification represents another important parameter. While well-defined scoring systems exist for PD-L1 (i.e., TPS and CPS), standardized scoring for TAMs and TILs based on immunohistochemistry is still lacking. For TILs, evaluation on H&E-stained sections using the human-adapted TIL scoring method is currently the most recommended approach.

For immunohistochemical evaluation, the use of whole slide imaging (WSI) and computer-assisted digital image analysis software is recommended to ensure objective, reproducible, and standardized quantification.

In conclusion, a prioritized roadmap for future research should focus on: identifying which veterinary tumors serve as the strongest comparative models, such as canine mammary tumors for breast cancer, oral melanoma for immune checkpoint blockade, and osteosarcoma for immuno-stromal atlases; and addressing the most critical knowledge gaps, including the need for standardized immunohistochemistry and TIL scoring, harmonization of PD-L1 assays, integration of ECM metrics, and the development of prospective biomarker-driven clinical trials.

## Glossary

CMC: Canine Mammary Carcinoma
CRC: Colorectal Carcinoma
CAFs: Cancer Associated Fibroblasts
CTLA-4: Cytotoxic T-Lymphocyte Antigen 4
DCs: Dendritic Cells
DFI: Disease-Free Interval
ECM: Extracellular matrix
EGFR: Epidermal Growth Factor Receptor
FMC: Feline Mammary Carcinoma
HSA: Visceral Hemangiosarcoma
NK: Natural Killer
PD-1: Programmed cell death protein 1
PD-L1/PD-L2: Ligand 1 and 2 of PD1
PMN-MDSCS: Polymorphonuclear myeloid-derived suppressor cells
STSs: Soft Tissue Sarcomas
TAMs: Tumor-Associated Macrophages
TILs: Tumor-Infiltrating Lymphocytes
TME: Tumor Microenvironment
Tregs: Regulatory T cells
TSS: Tumor-Specific Survival
MCT: Canine Mast Cell Tumors
OSCC: Oral Squamous Cell Carcinoma
OSA: Spontaneous Osteosarcoma
VCAN: Versican
